# Editorial

**Published:** 1892-08

**Authors:** 


### HOMŒOPATHIC PHYSICIAN,

A MONTHLY JOURNAL OF

HOMŒOPATHIC MATERIA MEDICA AND CLINICAL MEDICINE.

"If our school ever gives up the strict inductive method of Hahnemann, we are lost, and deserve only to be mentioned as a caricature in the history of medicine."—CONSTANTINE HERING.

Vol. XII. AUGUST, 1892. No. 8.

### EDITORIAL.

THE AMERICAN INSTITUTE OF HOMŒOPATHY.—In this number we give an exceedingly condensed report of the meeting this year at Washington of the American Institute of Homœopathy. The meeting was probably the largest ever held except that of last year in conjunction with the International Congress. As a consequence the business before the Convention was enormous, and could not all be satisfactorily finished. Our space is much too limited to publish any of the discussions, however interesting, and it has been a difficult task to decide what to give our readers and what to discard. It has cost some time to make a judicious selection. The work, such as it is, now lies before our readers, and they can get an idea of what they wish particularly to read and then seek it out in the Transactions officially published by the Institute.

Several brilliant and costly entertainments were given the Institute; among the most interesting being a visit to Mount Vernon upon a great river steamer specially chartered for the occasion, and a grand banquet on the opposite side of the river at Marshall Hall, followed by a *fête champêtre*. There were private receptions and a great public reception, and a visit to the President of the United States. All this was arranged by the Washington physicians, and it is probably the most costly and brilliant offering the Institute has ever received.

---

THE INTERNATIONAL HAHNEMANNIAN ASSOCIATION held a very successful meeting at Narragansett Pier. The papers were excellent and the discussions most interesting. It was also a harmonious meeting. Some unfortunate factions had arisen which threatened to consume time with profitless discussions that might have assumed a personal aspect. But they were all amicably adjusted by the good management of two or three earnest members, and so nothing occurred that would prevent the concentration of attention upon the subjects of medical practice for which they had come together. What these subjects were and what was said concerning them will be duly set forth in these pages from month to month.

In a short paragraph in "Notes and Notices" in last month's issue of this journal we gave the names of the officers of the Association. Among the censors we gave the name of Dr. Davis. This is a mistake, it should be Dr. B. L. B. Baylies, of Brooklyn.

---

THE DELAYED AUGUST NUMBER.—The editor feels that he owes an apology to the readers of this journal for its late appearance this month. Being severely overworked he took a much-needed vacation, and so all the work of the office was temporarily suspended.

